# A novel precision-serology assay for SARS-CoV-2 infection based on linear B-cell epitopes of Spike protein

**DOI:** 10.3389/fimmu.2023.1166924

**Published:** 2023-05-12

**Authors:** Samuel B. Lundin, Hanna Kann, Alma Fulurija, Björn Andersson, Sravya S. Nakka, Lars-Magnus Andersson, Magnus Gisslén, Ali M. Harandi

**Affiliations:** ^1^ Department of Microbiology and Immunology, Institute of Biomedicine, University of Gothenburg, Gothenburg, Sweden; ^2^ Biotome Pty Ltd, Perth, WA, Australia; ^3^ Biotome AB, Kullavik, Sweden; ^4^ School of Biomedical Sciences, Marshall Centre, University of Western Australia, Perth, WA, Australia; ^5^ Department of Infectious Diseases, Institute of Biomedicine, University of Gothenburg, Gothenburg, Sweden; ^6^ Region Västra Götaland, Sahlgrenska University Hospital, Department of Infectious Diseases, Gothenburg, Sweden; ^7^ Vaccine Evaluation Center, BC Children’s Hospital Research Institute, University of British Columbia, Vancouver, BC, Canada

**Keywords:** SARS-CoV-2, B-cell epitope, precision serology, Spike protein, cross-reactivity

## Abstract

**Introduction:**

The COVID-19 pandemic illustrates the need for serology diagnostics with improved accuracy. While conventional serology based on recognition of entire proteins or subunits thereof has made significant contribution to the antibody assessment space, it often suffers from sub-optimal specificity. Epitope-based, high-precision, serology assays hold potential to capture the high specificity and diversity of the immune system, hence circumventing the cross-reactivity with closely related microbial antigens.

**Methods:**

We herein report mapping of linear IgG and IgA antibody epitopes of the SARS-CoV-2 Spike (S) protein in samples from SARS-CoV-2 exposed individuals along with certified SARS-CoV-2 verification plasma samples using peptide arrays.

**Results:**

We identified 21 distinct linear epitopes. Importantly, we showed that pre-pandemic serum samples contain IgG antibodies reacting to the majority of protein S epitopes, most likely as a result of prior infection with seasonal coronaviruses. Only 4 of the identified SARS-CoV-2 protein S linear epitopes were specific for SARS-CoV-2 infection. These epitopes are located at positions 278-298 and 550-586, just proximal and distal to the RBD, as well as at position 1134-1156 in the HR2 subdomain and at 1248-1271 in the C-terminal subdomain of protein S. To substantiate the applicability of our findings, we tested three of the high-accuracy protein S epitopes in a Luminex assay, using a certified validation plasma sample set from SARS-CoV-2 infected individuals. The Luminex results were well aligned with the peptide array results, and correlated very well with in-house and commercial immune assays for RBD, S1 and S1/S2 domains of protein S.

**Conclusion:**

We present a comprehensive mapping of linear B-cell epitopes of SARS-CoV-2 protein S, that identifies peptides suitable for a precision serology assay devoid of cross-reactivity. These results have implications for development of highly specific serology test for exposure to SARS-CoV-2 and other members of the *coronaviridae* family, as well as for rapid development of serology tests for future emerging pandemic threats.

## 1 Introduction

In response to the COVID-19 pandemic, a large variety of SARS-CoV-2 serology tests have been developed. These assays use recombinant SARS-CoV-2 proteins, including Spike (S) and nucleoprotein (N) or protein subunits, including S1, S2 and receptor-binding-domain (RBD). There are also varying antibody classes targeted in these tests, with IgG antibody being the most common, while several tests detect IgM antibody, IgA antibody or all classes combined (1). Due to the urgency created by the pandemic, many tests that rapidly entered the market later had their approvals withdrawn due to insufficient validation and sometimes poor accuracy (2). A recent systematic review of currently available serology tests showed considerable variation in accuracy of SARS-CoV-2 serology tests with several presenting sub-optimal performance (1).

The COVID-19 pandemic further emphasizes the need for serology diagnostics with improved accuracy (3). Conventional serology assays based on recognition of entire proteins or subunits thereof are important public health tools for assessing infection exposure including in asymptomatic individuals. Nevertheless, these assays often suffer from sub-optimal specificity due to cross reactivity with closely related microbial antigens. Epitope-based, high-precision, serology assays hold potential to capture the high specificity and diversity of the immune system, hence circumventing the cross-reactivity with closely related microbial antigens.

Linear epitopes are not always suitable for analysis of antibody functions, but unlike conformational B-cell epitopes these methods are suitable for high-throughput analysis of linear epitopes (4, 5). This makes large-scale comprehensive discovery of linear B-cell epitopes cost-effective. In addition, the low cost of synthesis of peptides, makes them ideal as the basis for precision immunology diagnostics.

The aim of this study was to harness the power of precision serology to identify linear B-cell epitopes of SARS-CoV-2 S protein, that have the potential be used for development of highly specific serology diagnostics for SARS-CoV-2 infection. To meet this aim, we used peptide array technology to map linear B-cell epitopes spanning the entirety of SARS-CoV-2 S protein targeted by serum IgG and IgA antibodies of COVID-19 patients. Further, we substantiated the applicability of the lead linear B-cell epitopes by employment of the Luminex platform using a certified validation plasma sample set from COVID-19 patients. The Luminex results were well aligned with the peptide array results, and correlated with in-house and commercial immune assays for RBD, S1 and S1/S2 domains of protein S. In summary, we report a set of 3 highly discriminatory linear B cell epitopes of SARS-CoV-2 S protein of which 2 are exposed on the surface of the protein and 1 in the endo-domain. These results can contribute to the development of a precision antibody diagnostic test for SARS-CoV-2 infection.

## 2 Materials and methods

### 2.1 Patients and clinical samples

Patient samples were obtained from the Department of Infectious Diseases, Sahlgrenska University Hospital, Gothenburg, Sweden, between January and June 2020, as previously described (6, 7). Serum collection was done well before any SARS-CoV-2 vaccine was available, so none of the patients or controls had received prior vaccination for SARS-CoV-2. Briefly, patients displaying symptoms compatible with COVID-19 and PCR-verified as SARS-CoV-2 infected were included in the study. The peak severity COVID-19 symptoms varied from mild (score 2-3, neither treatment nor in-patient hospital care) to moderate/severe (score 4-6, requiring low-flow to high-flow nasal oxygen) according to the WHO Clinical Progression Scale (8). All blood samples for this study were retrieved before the start of treatment. Pre-pandemic serum samples were obtained from the same infectious disease unit and consisted of samples from patients admitted before the onset of the pandemic. In total, 40 SARS-CoV-2 infected patients were included; 18 of these were sampled between 1 and 13 days after symptom onset, and 22 were sampled between 14 and 51 days after symptom onset. In addition, 12 pre-pandemic healthy individuals were also included. The study was approved by the Swedish Ethical Review Authority (Registration number 2020–01771) and patients were included after written informed consent.

Samples used to validate linear epitopes were obtained from The National Institute for Biological Standards and Control, UK (NIBSC). Standardised CE-marked plasma samples (NIBSC code 20/B770) were used; 23 samples were from verified SARS-CoV-2-infected individuals, and 14 samples were from verified SARS-CoV-2 negative individuals. In addition, quality control samples with verified presence of anti-SARS-CoV-2 antibodies were used (NIBSC codes 20/B764 and 20/162).

### 2.2 Mapping of linear B-cell epitopes

IgG and IgA antibody-responses to SARS-CoV-2 S protein peptides were assayed using peptide array analysis. Medium-density arrays were created using laser jet-assisted on-chip synthesis technology. On these array chips, 1262 different 12-amino acid (12-mer) SARS-CoV-2 peptides were spotted onto each chip. Peptide sequences were from the Wuhan-Hu-1 strain of SARS-CoV-2 (NCBI accession NC_045512.2). The peptide sequences selected were sequential and overlapping and were spanning the entire amino acid sequence of protein S with a sequence overlap of 11 amino acids between each peptide.

To map antibody-binding to each peptide, each array was incubated with a 1/1000-dilution of a pool of 3 different serum samples from the same disease group, followed by washing and subsequent incubation by DyLight680-conjugated goat anti-human-IgG(Fc) and DyLight800-conjugated goat anti-human-IgA antibodies. Finally, fluorescence image scanning using a LI-COR Odyssey system, and subsequent digital image analysis was performed to detect antibody-binding to each of the peptides on the chip. Chip printing and antibody analysis was performed by PEPperPRINT (Heidelberg, Germany).

The background was detected by pre-incubating the array with secondary antibodies and measuring binding intensity to each peptide. The threshold for binding to a peptide by a serum sample was set to 3 x standard deviation (SD) above the average of the background, using log-transformed data. Sequence stretches with at least 3 consecutive peptides above background in at least two separate sample pools were considered as epitopes. Further, adjacent epitopes with overlapping borders were joined and regarded as one continuous epitope.

### 2.3 Validation and determination of frequency of use of epitopes

The primary identification of S protein epitopes was done using pooled serum samples. To validate the findings and determine the frequency of use of the strongest epitopes, 109 different protein S peptides were printed on new peptide arrays, and individual serum samples assayed for IgG and IgA antibody binding. This includes peptides from epitopes S_005, S_010, S_011, S_015, S_019, S_020 and S_021. In addition, peptides spanning the entire RBD, including S_006 through to S_009 were also added to the arrays. The arrays were produced and analysed as above, by PEPperPRINT (Heidelberg, Germany).

### 2.4 Detection of anti-SARS-CoV-2 antibodies by Luminex

Findings from subsequent B-cell epitope mapping experiments were further applied to Luminex xMap suspension array technology which allows simultaneous measurement of multiple antigens in a single well. Using the in-house Neutravidin modification of this multiplex serology method, we evaluated binding of anti-SARS-CoV-2 IgG antibodies to 12-mer peptides of S_010 (VRDPQTLEILDI, S protein position 575-586), S_019 (FKEELDKYFKNH, position 1147-1158) and S_021 (CCKFDEDDSEPV, position 1252-1263), to a control peptide from a non-epitope region of S protein (IFGTTLDSKTQS, position 104-115), and to recombinant RBD protein. In brief, biotin-coupled peptide or protein antigen were linked to a corresponding distinctly labelled subsets of Neutravidin-coated Luminex beads at 0.1 nmol peptides or RBD protein per 10^6^ beads. Subset of beads for background detection remained coated with Neutravidin with no further coupling. Background and antigen-coated beads were pooled just prior to a one-hour incubation with 1:200 diluted 23 positive and 14 negative serum samples from the certified SARS-CoV-2 serology assay validation sample set (NIBSC Verification panel, NIBCS, code: 20/B770). Particles were then washed with 1% BSA in PBS-Tween, followed by a 30-minute incubation with 1/50-dilution of Phycoerythrin labelled anti-IgG goat anti-human detection antibodies (Jackson ImmunoResearch, codes: 109-115-098). After another washing step, fluorescent signal was measured by the MagPix instrument. Assay read-out was reported in median fluorescent intensity units and within each plate well background bead values were extracted from those linked to the antigens. Certified sample set data for our antigens was compared with results from commercially produced gold-standard SARS-CoV-2 serology assays.

Biotinylated TTDS-peptides were produced by JPT Peptide Technologies GmbH (Berlin, Germany). Recombinant RBD antigen was sourced by Gothenburg University Mammalian Protein Expression core facility and biotinylated in-house with EZ-Link NHS-PEG4-Biotin (ThermoFisher, code: A39259).

## 3 Results

### 3.1 B-cell epitope mapping of SARS-CoV-2 protein S identified 21 linear epitopes

We first mapped all linear B-cell epitopes of the SARS-CoV-2 S protein by testing pooled sera for binding to S protein peptides in the peptide array. Using stringent cut-off criteria, we identified 21 linear epitopes of S protein that were used by at least two of the 7 serum sample pools tested. The average length of the epitopes were 17 amino acids. Of these, 90% were IgG antibody epitopes (n=19), 57% were IgA antibody epitopes (n=11), and 48% were both IgG and IgA antibody epitopes (n=10) (**Table 1**).

**Table 1:** Linear B-cell epitopes of SARS-CoV-2 proteins.

Epitope	Domain/Protein	Amino acid sequence	Start	End	Length	Class	Fold-change^1^	
							IgG	IgA
S_001	S1A	FNDGVY	86	91	6	IgA	n.a.^2^	0.6
S_002	S1A	CEFQFCNDPFLG	131	142	12	IgG	0.9	n.a.
S_003	S1A	KSWMESEFRVYSSANNCTFEYVSQPFLMDLEGKQGNFKNLREFVF	150	194	45	IgG, IgA	1.5	1.4
S_004	S1A	SALEPLVDLPIGINIT	221	236	16	IgG, IgA	5.6	2
S_005	S1A	KYNENGTITDAVDCALDPLSE	278	298	21	IgG, IgA	6.2	7.1
S_006	S1B/RBD	NVYADSF	394	400	7	IgG	2	n.a.
S_007	S1B/RBD	PDDFT	426	430	5	IgG	0.5	n.a.
S_008	S1B/RBD	FERDI	464	468	5	IgG	1.3	n.a.
S_009	S1B/RBD	NGVEGFNCYFP	481	491	11	IgG	2.1	n.a.
S_010	S1C	GVLTESNKKFLPFQQFGRDIADTTDAVRDPQTLEILD	550	586	37	IgG, IgA	3.9	2.3
S_011	S1D	PGTNTSNQVAVLYQDVNC	600	617	18	IgG	2.3	n.a.
S_012	S1D	IGAEHVNNSYECDIPI	651	666	16	IgG, IgA	0.7	1
S_013	S2-UH	ALTGIAVEQDKNTQE	766	780	15	IgG, IgA	3.5	1.4
S_014	S2-UH	KTPPIKDFGGFNFSQILPD	790	808	19	IgG	1.4	n.a.
S_015	S2-FP	SKRSFIEDLLFN	813	824	12	IgG	1.9	n.a.
S_016	S2	QYGDCLGDI	836	844	9	IgG	1.7	n.a.
S_017	S2-HR-1	SRLDKVEAEVQID	982	994	13	IgG, IgA	6.6	3.7
S_018	S2-BH	FPREGV	1089	1094	6	IgA	n.a.	1.2
S_019	S2-HR2	NNTVYDPLQPELDSFKEELDKYF	1134	1156	23	IgG, IgA	2.6	1.8
S_020	S2-HR2	PDVDLGDISGINASVVNIQKEIDRLNEVAKNLNESLIDLQELGKYEQ	1162	1208	47	IgG, IgA	2	1.4
S_021	S2-CT	CSCGSCCKFDEDDSEPVLKGVKLH	1248	1271	24	IgG, IgA	2.8	0.8

^1^Fold-change: ratio for the response of SARS-CoV-2-infected vs non-infected sample pools. Samples were pooled (n = 3 samples per pool), 7 pools of samples from infected individuals were compared to one pool of samples from uninfected (pre-pandemic) individuals.

^2^n.a, Not applicable. The ratio of response was calculated only for the relevant antibody class(es).

**Figure 1:**
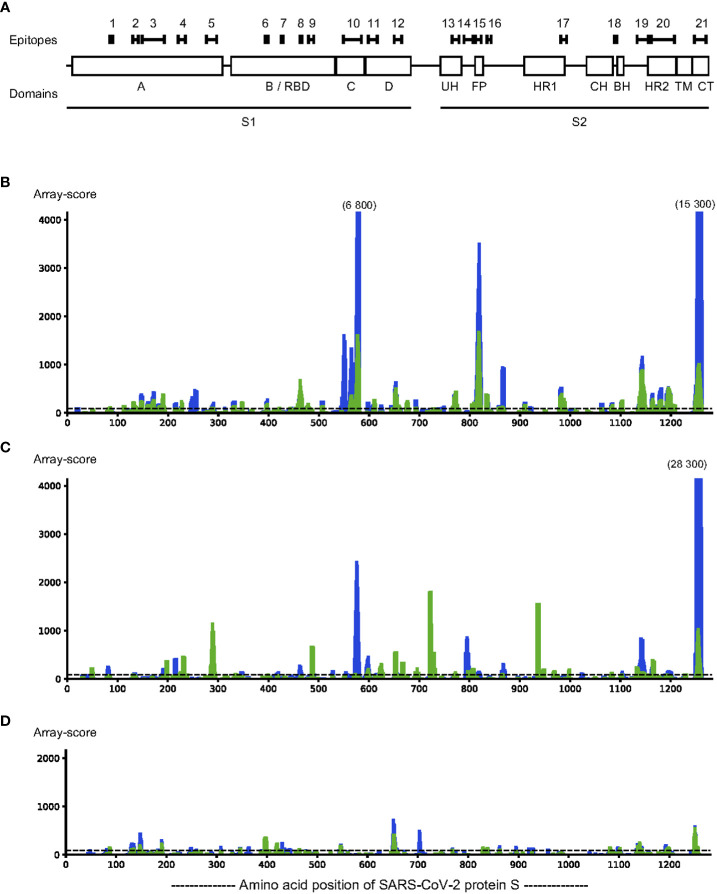
Linear epitopes were identified in SARS-CoV-2 protein S by assaying IgG binding to sequential 12-mer peptides, printed with 11 amino acid sequence overlap, using peptide arrays (n=1262 peptides). Pooled sera (n=5 sera per pool) from SARS-CoV-2 infected individuals and uninfected individuals were incubated on the arrays and antibody binding measured. Epitopes were defined as stretches of at least 3 consecutive peptides with a response above background in at least two different samples pools. Locations of the epitopes indicated over the sequence of protein S are indicated with black bars, above the locations of each subdomain **(A)**. Binding strength of IgG (blue) and IgA (green) is indicated over the sequence of protein S **(C, D)**. Representative pools from SARS-CoV-2 infected **(B, C)** and from pre-pandemic samples of uninfected controls **(D)** were incubated on the arrays; IgG binding to each peptide was detected with DyLight680-labelled anti-human IgG antibodies followed by scanning to detect fluorescence. The fluorescence signal (y-axis) for binding for each peptide along the amino acid position of protein S (x-axis) is expressed as the rolling average of the three adjacent peptides of each amino acid position. The background level (y=90), determined by incubating anti-human IgG with the array in the absence of serum, is indicated as a dotted horizontal line. The y-axis is clipped at 4000, any peak value above 4000 is indicated with the maximum levels above such peaks.

According to S protein domain boundaries described by Barnes et al. (9), we identified epitopes both in the S1 and S2 domains (**Figure 1** and **Table 1**). The S1 domain had 12 epitopes (S_001 – S_012), located in all subdomains (S1^A-D^), including 4 epitopes in the receptor binding domain (S1^B^/RBD) (S_006 – S_009). There were 9 epitopes in the S2 domain (S_013 – S_021), spanning sub-domains S2^UH^, S2^FP^, S2^HR1^, S2^BH^, S2^HR2^, and S2^CT^ (**Figure 1** and **Table 1**). For 11 of these 21 epitopes, at least one amino acid residue in, or very close to, the epitope harbours a mutation frequently observed in the SARS-CoV-2 variants of interest. These affected epitopes include S_007 (K417N of Beta and Omicron and K417T of Gamma), epitope S_009 (T478K of Delta, E484K of Beta and Gamma, E484A of Omicron, and N501Y of Alpha, Beta and Gamma), S_010 (A570D of Alpha), S_011 (D614G of Alpha, Beta, Gamma, Delta and Omicron) and S_012 (H655Y of Gamma and Omicron).

### 3.2 Sera from individuals never exposed to SARS-CoV-2 have IgG and IgA antibodies to a large fraction of S protein linear epitopes

To identify areas of S protein that could be used for accurate assessment of antibody-responses in infected vs uninfected individuals, and thereby identify current or past SARS-CoV-2 infection, we tested a group of serum samples taken before the pandemic (pre-COVID-19 samples). We found that in as much as 29% of all S protein peptides (n=370 out of 1262 peptides) there was a response above the background cut-off in either IgG or IgA antibodies in these pre-COVID-19 samples.

Within the identified epitopes, the pre-COVID-19 samples had an IgG antibody response higher than the median of infected samples in 14% of the epitopes (n=3) and an IgA antibody response higher than the median of infected samples in 29% (n=6) of the epitopes. The epitopes for which there is a response only in SARS-CoV-2-infected patients and not in pre-pandemic samples constitute only 47% of IgG antibody epitopes and 25% of IgA antibody epitopes of S protein (**Table 1**). These findings highlight that there is a real risk for creating false-positive test results unless serology development takes cross-reactive epitopes into account.

### 3.3 Lead B-cell linear epitopes from protein S are useful for diagnosis when analysed individually

**Figure 2:**
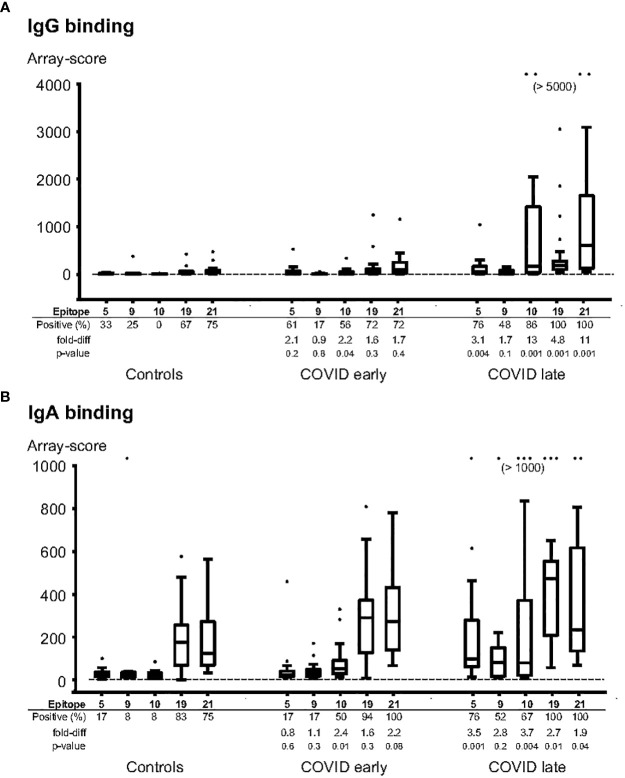
Individual patient samples tested for binding of IgG **(A)** and IgA **(B)** to selected epitope peptides. The most diagnostic peptides from epitopes 5, 9, 10, 19 and 21 were synthesized on peptide arrays, and the arrays were incubated with individual serum samples from pre-pandemic controls (n=12), COVID-patients sampled 1-13 days after symptom onset (“COVID early”, n=18), or COVID-patients sampled 14-50 days after symptom onset (“COVID late”, n=22). The data is expressed in box-whisker plots, where the median (horizontal line inside the box), the interquartile range (top and bottom of the box), and 1.5 x the interquartile range (the top and bottom whiskers) are indicated. Any outliers, outside the 1.5x interquartile range, are indicated as individual dots. The cutoff level for a positive response (mean + 3 x Standard deviation of the background) is indicated as a horizontal line. Positive (%): the frequencies of samples with a positive response for each peptide; fold-diff: the fold-difference between the median for each peptide for the COVID group samples vs the Control group samples; p-value: Mann-whitney p-value for difference for each peptide between the COVID group samples and the Control group samples.

Guided by the results from the primary screening phase, we analysed individual COVID-19 patient sera along with pre-COVID-19 control serum samples using new peptide arrays comprised of discriminatory linear peptides identified in the primary screening of linear B-cell epitopes of S protein. These arrays contained peptides covering the most strongly reactive epitopes from the screening phase, in addition to overlapping peptides covering the RBD (n=109 peptides in total). We tested the ability of these peptides to diagnose SARS-CoV-2 infection by assessing IgG and IgA antibody-binding to each peptide for samples from SARS-CoV-2 infected individuals obtained at 14 days or more after onset of symptoms (n = 22) and from samples obtained before the pandemic (n = 12). To assess the levels of cross-reactivity to each peptide, we calculated the Receiver Operating Characteristic Area Under the Curve (AUC) for antibody-responses to each of these peptides when comparing SARS-CoV-2-infected with pre-pandemic samples. Several peptides that could be used to discriminate IgG antibody-responses of SARS-CoV-2 infected samples from pre-pandemic controls were found, with an AUC of at least 0.90 for 2 peptides, and an AUC of at least 0.80 for 8 peptides. These highly discriminatory peptides belonged to epitopes S_005, S_010, S_S019 and S_021 (**Table 2**). The strongest responses were seen to epitopes S_010 and S_021 (**Figures 1**, **2**); with 13-fold and 11-fold difference between the median of SARS-CoV-2 infected samples and controls, respectively.

**Table 2:** The most discriminatory peptides for SARS-CoV-2 infection.

Domain	Epitope	Amino acid sequence	Position^1^	AUC IgG^2^	AUC IgA^3^
S1C	S_010	VRDPQTLEILDI	575	0.94	0.80
S1C	S_010	TDAVRDPQTLEI	572	0.90	0.78
S2-CT	S_021	CCKFDEDDSEPV	1252	0.88	0.71
S1C	S_010	PFQQFGRDIADT	560	0.88	0.58
S2	S_019	QPELDSFKEELD	1141	0.84	0.77
S2-CT	S_021	FDEDDSEPVLKG	1255	0.83	0.87
S1A	S_005	YNENGTITDAVD	278	0.81	0.87
S1C	S_010	QFGRDIADTTDA	563	0.80	0.73
S1C	S_010	ADTTDAVRDPQT	569	0.73	0.80

^1^ Position: The amino acid position of each peptide within the protein from where it originates.

^2,3^ AUC: Discrimonatory capacity (Receiver Operating Characteristic Area Under the Curve) for antibody-levels to each peptide, comparing samples from SARS-CoV-2-infected (n = 22) sampled at least 14 days after symptom onset vs non-infected pre-pandemic (n = 12) individuals. Samples were tested individually, in peptide arrays containing 213 different SARS-CoV-2 peptides. Only peptides with an AUC of 0.80 or above are shown.

For IgA antibody responses, there were 4 peptides with an AUC of at least 0.80 but none with an AUC of 0.90 or above (**Table 2**). The IgA antibody-discriminatory peptides belonged to epitopes S_005, S_010 and S_021 (**Table 2**). The IgA antibody response was generally lower than the IgG antibody response, with a maximal fold-change of 3.5 (epitope S_005) and 3.7 (S_010) compared to controls.

Epitope S_010 had lowest percentage of positive samples among controls (0% for IgG antibody and 8% for IgA antibody). This was also the epitope with the strongest response in samples taken before day 14 of symptom onset - 2.2 and 2.4-fold increase compared to controls for IgG and IgA antibodies, respectively.

Among 4 most discriminatory peptides, only S_010 harbours one of the main mutations of any of the variants of concern (A570D of Alpha); however, the two most diagnostically discriminatory peptides of S_010 are located distal to position 570 (peptides TDAVRDPQTLEI, at position 572-583, and VRDPQTLEILDI, at position 575-586, **Table 2**). Thus, the most discriminatory peptides of protein S may likely be used as universal serology diagnostics markers for exposure to all major SARS-CoV-2 variants.

### 3.4 Most key linear S protein epitopes are exposed on the surface of the protein, while S_021 is located inside the protein

**Figure 3:**
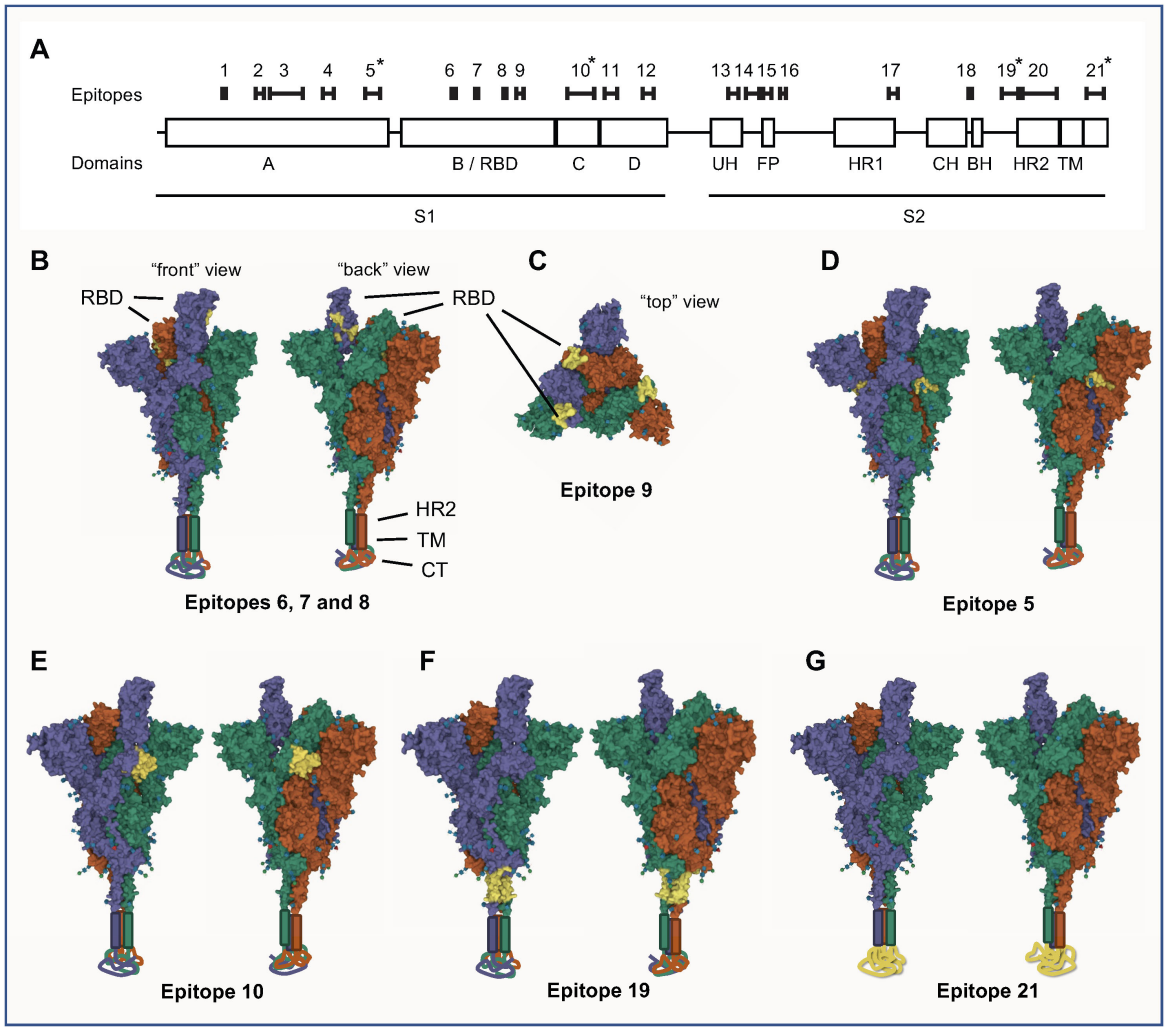
Linear epitopes were identified in SARS-CoV-2 S protein by assaying IgG binding to sequential 12-mer peptides, printed with 11 amino acid sequence overlap, using peptide arrays. Pooled sera (n=7 different pools, n=5 sera per pool) from SARS-CoV-2 infected individuals were incubated on the arrays and IgG binding measured. Epitopes were defined as stretches of at least 3 consecutive peptides with a response above background in at least two different samples pools. **(A)** Schematic view of the location and length of the identified epitopes along a linear representation of SARS-CoV-2 S protein. Epitopes are numbered 1-21, and are shown in sequence alignment with domains and subdomains of S protein. The most consistently diagnostic epitopes (5, 10, 19 and 21) are indicated by asterisks (*). **(B-G)** 3D-representations of the locations of the most noteworthy linear epitopes on the S protein trimer. Space-filling views of S protein in a “one RBD-up” conformation (PBD-accession 7KRR) is shown, where the three different S protein monomers are shown in purple (RBD-up), green and red (both RBD-down), respectively. Each model is viewed from two different angles (“front” and “back”); in **(C)** it is also viewed from above (“top”). For completeness, visual representations of HR2, TM and CT, all absent from the 7KRR model, have been added manually to the base of the trimer. Epitopes are indicated in yellow on all three S protein monomers. **(B, C)** Epitopes of the RBD (epitopes 6, 7, 8 and 9) are indicated in yellow. Epitopes 6-8 are only visible in the RBD-up state **(B)** while epitope 9 is visible in both states **(C)**. The most diagnostic epitopes are shown in **(D)** – epitope 5; **(E)** – epitope 10; **(F)** – epitope 19; and **(G)** - epitope 21. Epitope 21 spans most of the CT subdomain, which is structurally undetermined and located below the virus envelope inside the virion. 3D-representations were made with the Mol* viewer software.

To assess whether these important epitopes are exposed on the native protein S, we analysed their location on a 3D model of the protein S trimer using the Mol* viewer software (10). The RBD of S protein can take two different states/conformations, either “RBD-up”, where the RBD is exposed for facilitated receptor binding, or “RBD-down” where the RBD is not exposed to the same degree (11). Of the epitopes located in RBD domain (S_006 – S_009, **Figure 3A**), only S_009 was exposed in both the RBD-up and RBD-down state, while epitopes S_006 – S_008 were concealed in the RBD-down state and only exposed in the RBD-up state (**Figures 3B, C**). The latter three epitopes were located very close to each other and may be part of a joint conformational epitope.

Both epitopes S_005 and S_010 were, to a large extent, modelled to be surface exposed very close to the RBD, both in the RBD-up and RBD down state (**Figures 3D, E**). Interestingly, despite located on the proximal (S_005) and distal (S_010) side of RBD on the amino acid chain, these two epitopes were located just adjacent to each other on the trimer surface; S_005 on one S protein monomer was located next to the S_010 of the adjacent S protein monomer (**Figures 3D, E**). There was no correlation between the antibody-levels to these two epitopes in individual patient samples (spearman coefficient: 0.04), so despite their close spatial proximity they are unlikely to belong to the same conformational epitope.

Epitope S_019 was found to be located in the basal part of the protein S trimer, just outside the viral envelope (**Figure 3F**). The epitope S_021, the epitope with overall strongest antibody binding for both IgG and IgA antibodies, was found to be in the C-terminal stretch located internally in the virion, inside the viral envelope (**Figure 3G**). This section of protein S is not structurally well-determined and therefore likely in an intrinsically disordered state (12).

### 3.5 Lead protein S linear peptides can identify SARS-CoV-2 antibodies in a Luminex-based assay

To validate the peptide array findings and assess whether anti-peptide antibodies can be detected using a method more relevant for conventional serology diagnostics, we set up a Luminex-based assay and tested lead S protein peptides. Biotinylated peptides from epitope S_010, S_019 and S_021, or biotinylated recombinant RBD were bound to neutravidin-coupled Luminex beads and IgG antibody levels in clinical samples were analysed.

**Figure 4:**
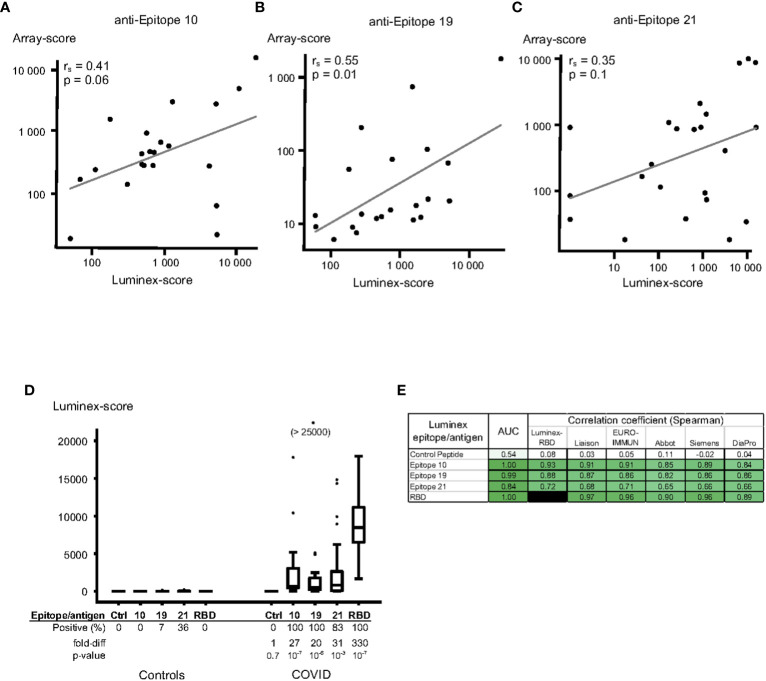
Luminex assay for quantitation of IgG anti-SARS-CoV-2 protein S peptides/antigens using a validation set of plasma sampes. Protein S peptides or recombinant receptor-binding domain (RBD) of protein S were linked to Luminex beads using Neutravidin – biotin, followed by incubation with plasma samples and fluorescent anti-IgG antibodies. **(A-C)** Correlations between Luminex and array-data for epitope 10 **(A)**, epitope 19 **(B)** and epitope 21 **(C)**. Results from Luminex assays (x-axis) were compared to results from peptide array assays (y-axis) for the same samples; the data is plotted on a log-scale. The Spearman correlation coefficients (r_s_), p-values and linear regression line for each peptide correlation are indicated in the figures. **(D, E)** Validation of the Luminex assay using certified anti-SARS-CoV-2 serology verification samples from uninfected (“Controls”, n=14) and infected (“COVID”, n=23) individuals. **(D)** Peptides from epitopes 10 (peptide VRDPQTLEILDI), 19 (FKEELDKYFKNH) and 21 (FDEDDSEPVLKG), and a control peptide (IFGTTLDSKTQS, “Ctrl”), as well as recombinant RBD were tested using Luminex. The data is expressed in box-whisker plots, where the median (horizontal line inside the box), the interquartile range (top and bottom of the box), and 1.5 x the interquartile range (the top and bottom whiskers) are indicated. Any outliers, outside the 1.5x interquartile range, are indicated as individual dots. **(E)** The discriminatory capacity of the Luminex-methods were tested by calculating the AUC (ROC-AUC) values between certified Controls and SARS-CoV-2 infected cases. The correlation of the Luminex methods and each of different commercial SARS-CoV-2 protein S IgG serology assays was assessed, and Spearman correlation coefficients (r_s_) displayed. The colour of each cell is ranging from white for a value close to 0, and intense green for values close to 1.00.

We first examined the correlation between Luminex scores and peptide array results for the same peptides. There were positive, but relatively modest, correlations between these assays; the spearman correlation coefficient comparing array and Luminex results was 0.41 (p = 0.06) for epitope S_010, 0.55 (p = 0.01) for epitope S_019 and 0.35 for epitope S_021 (p = 0.1) (**Figures 4A–C**).

To assess the diagnostic capacity of individual peptides in the Luminex assay, we tested a set of certified SARS-CoV-2 serology verification samples (NIBSC), unrelated to the samples used to identify the peptides in the peptide array experiments. Peptides from all three epitopes gave a strong IgG antibody response in the certified COVID-19 plasma samples (27-, 20- and 31-fold increases over control samples for epitopes 10, 19 and 21, respectively, **Figure 4D**). The diagnostic capacity of all epitope peptides was excellent in this validation set (AUC 1.00, 0.99 and 0.84 for the three peptides, **Figure 4E**), and the control peptides did not show any IgG antibody binding (**Figure 4D**). The three epitopes also exhibited a strong correlation to the anti-RBD response (spearman coefficient 0.93, 0.88 and 0.72, respectively; **Figure 4E**). In order to bench-mark our approach to a gold-standard, we then compared our results to those of widely used commercial anti-SARS-CoV-2 IgG antibody ELISAs made available through NIBSC, UK (tests produced by Liaison, EURO-IMMUNE, Abbot, Siemens and DiaPro). The Luminex responses significantly correlated with results from these ELISA tests (spearman coefficients 0.84-0.91 for S_010, 0.82-0.87 for S_019 and 0.65-0.71 for S_021; **Figure 4E**).

## 4 Discussion

Herein, we employed peptide microarray technology and a Luminex-based assay along with serum samples retrieved from COVID-19 patients, COVID-19 naïve individuals (pre-pandemic samples) and a set of certified standard COVID-19 plasma samples to profile linear B-cell epitopes of SARS-CoV-2 S protein and define highly discriminatory epitopes. None of the samples tested were from individuals that had received prior vaccination for SARS-CoV-2. We report 21 different B-cell linear epitopes of SARS-CoV-2 S protein, only 4 of which were found highly discriminatory and hence useful for accurate sero-diagnosis of SARS-CoV-2 infection. Our modelling analysis revealed that 3 of these key epitopes (S_005 - KYNENGTITDAVDCALDPLSE, positions 278-298 in subdomain S1A; S_010 - GVLTESNKKFLPFQQFGRDIADTTDAVRDPQTLEILD, positions 550-586 in subdomain S1C; and S_019 - NNTVYDPLQPELDSFKEELDKYF, positions 1134-1156 in subdomain S2-HR2) are exposed on the surface of the protein and one (S_021 - CSCGSCCKFDEDDSEPVLKGVKLH, positions 1248-1271 in subdomain S2-CT) is located in the endo-domain of the protein.

Our results are, by and large, in line with recent studies that examined the B-cell linear epitopes of SARS-CoV-2 S protein, and also extend these findings. Ladner et al. reported a detailed profile of B-cell linear epitopes of SARS-CoV-2 S and N proteins using a peptide library of 30-mer peptides (13). They identified 3 highly used epitopes in S protein (positions 560-572, 819-824 and 1150-1156). We identified these regions as epitopes in our study, and they are included in the epitopes S_010, S_015, and S_019 (**Table 1**). However, using our approach, these particular epitope stretches are not among the most highly discriminatory epitopes (**Table 2**). Further, Shrock et al. reported a comprehensive mapping of SARS-CoV-2 antibody responses using the VirScan technology, which uses a library of 50- and 20-mer peptides spanning the entire proteome of SARS-CoV-2 (14). They propose a 3-peptide assay for accurate SARS-CoV-2 sero-diagnosis – two epitopes of S protein (positions 810-830 and 1146-1166) and one epitope in N protein (positions 386-406). These regions are defined in our study as part of epitopes S_015 and S_019. However, neither of those peptides are among the ones we identified as the most highly discriminatory (**Table 2**). Of note, these two peptide mapping reports were generated using peptide libraries with longer peptides (20-, 30- or 50-mer) than those used in our present study (12-mer). Further, the previously reported studies analysed samples in suspension while we used peptides immobilised onto an array surface. We suggest that the discrepancies with our study may be due to in which way the peptides are presented to the antibodies (in suspension/using phage display/on an array surface). We argue that our approach has an advantage for diagnostics as most immunoassays used for serology analysis utilise antigens immobilised on to a surface. We also argue that an approach that uses shorter peptides for discovery of markers for diagnosis is desirable in order to minimize the risk of containing cross-reactive sequences. This notion is supported by a considerable reactivity to SARS-CoV-2 peptides we observed in pre-pandemic samples (**Table 1** and see (14).

Musico et al, using medium-density peptide arrays made by the same technology as in the present study, reported 12 different 15-mer linear B-cell epitopes that may be useful for diagnosis of SARS-CoV-2 exposure (15). Although 3 of those 12 peptides were among the epitopes we identified (epitopes S_001, S_008, S_009), none of these were found among those we identified as the most discriminatory epitopes (**Table 2**). Mishra et al. used high-density peptide arrays, by which they identified 11 putatively discriminatory linear epitopes of SARS-CoV-2 S protein (16). Our results overlap with theirs in that we identified all their 11 reported epitopes except two (SP4, SP5, located in the region 671-706 of S protein). Two out of the 4 epitopes we identified as being most highly discriminatory were identified by Mishra et al. - the epitopes we designated S_010 was spanning their epitopes SP1 and SP2, and the epitope we designated S_019 was identified as SP10.

Taken together, all S protein epitopes we identified were partially or fully overlapping with recently published epitopes (17–19), and our criteria identified 50% of S protein epitopes that were reported in the IEDB database (20). Of note, two of the identified epitopes (S_010 and S_015) have been reported as virus neutralising epitopes (17). This finding can pave the way for development of low-cost peptide-based precision diagnostics for neutralising antibodies.

It was somewhat un-expected that epitope S_021 showed strongest antibody-binding (**Figures 1B, C**, **2A**) of all the epitopes we identified. This epitope is positioned in the endo-domain of protein S, which is located inside the virion, on the basal side of the viral envelope (**Figure 3G**). Interestingly, S protein endo-domains are important for viral fusion and for cell-cell fusion/formation of syncytia both in SARS-CoV-2 and in other Coronaviruses (21). In our hands, there was evidence of pre-existing IgA anti-S_021 antibodies (**Figure 2B**). This indicates that S_021 may have some degree of relevant cross-reactivity to other coronaviruses. Thus, prior exposure to seasonal coronaviruses may have primed the response to this epitope, and thereby explain the strong responses observed after SARS-CoV-2 infection.

Some interesting aspects were revealed by our 3D structural modelling of the highly discriminatory linear B-cell epitopes. Epitope S_010 has been described as being part of neutralising epitope (17). Since it is located next to the base of RBD and is surface-exposed both in the RBD-down and RBD-up states (**Figure 3E**), it is possible that its neutralising activity is mediated by its ability to interfere with switching from the RBD-down to the RBD-up states. This warrants further study.

Epitope S_009 (position 481-491) is surface exposed in both RBD-down and RBD-up states (**Figure 3C**). This epitope is partially overlapping with the binding surface of ACE2 (22), and is therefore a putative neutralising epitope. This is further supported by the fact that the E484 residue, located in this epitope, is a key mutation site in most variants of concern and important for immune escape. It was shown that a mutation in this residue can greatly reduce the neutralisation ability of sera from SARS-CoV-2 infected individuals (23).

The Luminex experiments substantiated the usefulness of the discriminatory peptides with a test platform more suitable for conventional serology use. Nonetheless, the correlations between results from peptide array and Luminex were relatively modest (**Figures 4A–C**). This may, at least partly, be explained by the different linking chemistries used for the peptide array and Luminex beads. This warrants further exploration of other linking chemistries and/or other assay platforms (ELISA, MSD etc) for peptide-based serology assays.

The high discriminatory capacity reached with the certified validation sample set is very encouraging. To pinpoint the accuracy in a clinical setting, larger sample size, especially from pre-pandemic SARS-CoV-2 negative controls, need to be tested.

It was not within scope of this study to identify differences in epitope use between vaccinated and non-vaccinated individuals. Further studies are needed to investigate whether inclusion of certain non-vaccine epitopes in the serology test could be used to distinguish these responses. Further, since 11 of the 21 identified epitopes contained at least one amino acid affected by mutations of VOCs, it is possible that some of these epitopes may be suitable for differential serology diagnostic of different VOCs. It is envisaged that employment of precision serology-based approach would add value for rapid development of serology tests for emerging pandemic threats.

In conclusion, we report a comprehensive linear B-cell epitope map of SARS-CoV-2 S protein, consisting of 21 epitopes. Within this map, we identify 3 highly discriminatory linear B-cell epitopes suitable as antigens in antibody/serology tests for SARS-CoV-2 infection. These results have implications for development of precision serology tests for sero-diagnosis of SARS-CoV-2 infection as well as for infections with other coronaviruses.

## Data availability statement

The raw data supporting the conclusions of this article will be made available by the authors, without undue reservation.

## Ethics statement

The studies involving human participants were reviewed and approved by Swedish Ethical Review Authority (Registration number 2020–01771). The patients/participants provided their written informed consent to participate in this study.

## Author contributions

SL: development of concept and design; collection, analysis and interpretation of peptide microarray and peptide structure data; preparation of figures and manuscript writing; funding acquisition. HK: Luminex method development, experimentation, data collection and data analysis; intellectual input; critical revision of the manuscript. AF: conceptualization, intellectual input and critical revision of the manuscript. BA: methodology, data analysis and statistics, manuscript review. SN: preparation of biological samples, intellectual input, manuscript review. L-MA: conceptualization, resources, manuscript review, supervision, funding acquisition. MG: resources, manuscript review, supervision, funding acquisition. AH: development of concept and design, project administration, resources, critical manuscript revision, supervision, funding acquisition. All authors contributed to the article and approved the submitted version.
